# HIV-1-associated PKA acts as a cofactor for genome reverse transcription

**DOI:** 10.1186/1742-4690-10-157

**Published:** 2013-12-17

**Authors:** Charline Giroud, Nathalie Chazal, Bernard Gay, Patrick Eldin, Sonia Brun, Laurence Briant

**Affiliations:** 1 Centre d’étude d’agents Pathogènes et Biotechnologies pour la Santé (CPBS)-CNRS UMR 5236, Université Montpellier 1,2, 1919 route de Mende, Montpellier, cedex 2 34293, France

**Keywords:** HIV, Reverse transcription, cAMP-dependent kinase

## Abstract

**Background:**

Host cell proteins, including cellular kinases, are embarked into intact HIV-1 particles. We have previously shown that the Cα catalytic subunit of cAMP-dependent protein kinase is packaged within HIV-1 virions as an enzymatically active form able to phosphorylate a synthetic substrate *in vitro (Cartier et al. J. Biol. Chem. 278:35211 (2003))*. The present study was conceived to investigate the contribution of HIV-1-associated PKA to the retroviral life cycle.

**Results:**

NL4.3 viruses were produced from cells cultured in the presence of PKA inhibitors H89 (H89-NL4.3) or Myr-PKI (PKI-NL4.3) and analyzed for viral replication. Despite being mature and normally assembled, and containing expected levels of genomic RNA and RT enzymatic activity, such viruses showed poor infectivity. Indeed, infection generated reduced amounts of strong-strop minus strand DNA, while incoming RNA levels in target cells were unaffected. Decreased cDNA synthesis was also evidenced in intact H89-NL4.3 and PKI-NL4.3 cell free particles using endogenous reverse transcription (ERT) experiments. Moreover, similar defects were reproduced when wild type NL4.3 particles preincubated with PKA inhibitors were subjected to ERT reactions.

**Conclusions:**

Altogether, our results indicate that HIV-1-associated PKA is required for early reverse transcription of the retroviral genome both in cell free intact viruses and in target cells. Accordingly, virus-associated PKA behaves as a cofactor of an intraviral process required for optimal reverse transcription and for early post-entry events.

## Background

Reverse transcription is a key step in early replication of the human immunodeficiency virus type 1 (HIV-1) (for review see [1]). Once entered in the cell cytoplasm, the single-stranded retroviral RNA genome is converted into a double-stranded DNA molecule capable to integrate into the host genome. The reverse transcription is primed by a human tRNA^Lys3^ primer packaged in virions that anneals to the primer binding site (PBS) and to an 8 nucleotide base-paired sequence upstream of the PBS known as the primer activation signal. The elongation reaction is catalyzed by the viral reverse transcriptase (RT), a heterodimeric enzyme formed of the p66 subunit that displays RNA-dependent DNA polymerase and RNase H activities, associated with the p51 subunit devoid of enzymatic activity. The minus-strand DNA is first synthesized by copying a short sequence at the 5′ end of the RNA genome complementary to the R and U5 regions. This step gives rise to the minus-strand strong stop DNA ((-)ssDNA)). Concomitantly with the elongation reaction catalyzed by the RT, the genomic RNA is degraded by RNase H activity of RT. Then the template is transferred to the 3′ end of the genome in a reaction facilitated by annealing of the complementary R regions. This strand transfer allows the regeneration of the long terminal repeat sequences. Synthesis of the plus-strand DNA ((+)ssDNA) is initiated from the PPT fragment of genomic RNA and is extended by RT using a template consisting of (-)ssDNA. The final full length double-stranded (FL) DNA is generated after a second template switch with each strand using other as a template.

In infected cells, FL DNA synthesis is assisted by HIV-1 encoded proteins present in the reverse transcription complex (RTC). Among them, the nucleocapsid protein (NC) is required for the initiation, chaperoning strand transfer and spatio-temporal control of the reaction [2,3]. Expression of Tat and physical contacts of RT with integrase and Vpr are also required for efficiency initiation of cDNA synthesis [4-6]. Finally, Nef and Vif proteins enhance RT affinity for the RNA genome and allow temporal regulation of RNA dimerization during early RT reaction [7-10]. In spite of the amount of information available on the mechanisms of HIV-1 DNA synthesis, many of the post-entry events that regulate the reverse transcription of HIV-1 genomic RNA remain unsolved (reviewed in [11]). As an example, the exact place where RT occurs in the infected cell cytoplasm, as well as the context in which proviral DNA is produced remains unknown. *Ex vivo* endogenous reverse transcription assays (ERT) helped clarifying some aspects of the reverse transcription process. First, this experimental approach pointed that the RT reaction is initiated and can proceed in the intact viral particle [12]. Indeed, (-)ssDNA, first-strand transfer and full-length minus strand products are detected in intact core fractions following incubation of the viral particle with deoxynucleotides (dNTPs). Hence, the biochemical activities necessary for the completion of early HIV-1 plus strand synthesis to the second-strand transfer step are retained in the core particle. Second, a direct correlation exists between HIV-1 capsid core organization and reverse transcription. This concept is supported by the critical morphological modifications of the retroviral core, including dissolution of the p24-shelled viral core and absence of the core-envelope linkage region, observed following intravirion reverse transcription [13]. Moreover, recent quantification of RT DNA intermediates produced in assembled HIV-1 particles revealed that ERT activity is increased in viral particles incubated with mild concentration of detergent [14]. Disruption of the HIV-1 core, by mean of high concentrations of detergents was, in contrast, coincident with the loss of the ERT activity of virions, particularly with a significant reduction of late reverse transcription products synthesis [14]. This aspect is reminiscent of the inability of p24 mutants with alteration in core stability to perform reverse transcription [15]. The persistence of an assembled core structure may therefore benefit to the reverse transcription process. Third, it is also clear that reverse transcription most likely involves host cell cofactors. The significant enhancement of first-strand transfer and late RT products synthesis evidenced in ERT assay performed in the presence of cell extracts supports the contribution of cell encoded proteins in assisting the reverse transcription reaction [14,16]. Some of the cofactors required for proviral DNA synthesis are recruited to the reverse transcription complex (RTC) independently of interactions with HIV-1 RT. This is illustrated by the capacity of the survival motor neuron (SMN)-interacting protein 2 (Gemin2), an HIV-1-integrase binding protein, to enhance the assembly of RT on viral RNA *in vitro* and to stimulate (-)ssDNA production *in vivo*[17,18]. A similar contribution was also reported for integrase interactor 1 (INI1, hSNF5) [19,20], sin3A-associated protein (SAP18) and histone deacetylase 1 (HDAC1) [21]. Recruitment to the RTC of the human topoisomerase Topo1 and of the DHX9 RNA helicase packaged into HIV-1 particles through interaction with NC and Gag respectively, is also required for optimal reverse transcription reaction [22,23]. Recently, two subunits of the eukaryotic elongation factor 1, eEF1A and eEF1G interacting with p51 RT subunit and the retroviral integrase were reported to assist completion of reverse transcription by stabilizing the RTC in the cytoplasm of the infected cells [24]. A number of cofactors assisting early steps of HIV-1 replication were also identified by recent genomic screens [25-27]. Among them, RNA helicase and DNA repair activities were shown to physically interact with RT and to assist early post entry steps. Some of these studies, together with two-hybrid experiments, also pointed the requirement for A-kinase anchoring protein (AKAP) proteins, a family of proteins ensuring localization of the cAMP-dependent protein kinase to cellular membranes, to assist early HIV-1 DNA synthesis. Indeed, both AKAP13 knock down and inhibition of AKAP149/RNase H domain of RT interaction were reported to be deleterious for optimal reverse transcription [25,28]. The exact role of AKAPs in reverse transcription still remains unelucidated. Of note, in addition to RT cellular cofactors, host proteins counteracting HIV-1 reverse transcription have also been identified in the cell cytoplasm. The category of proteins is mainly represented by APOBEC3G. This cytidine deaminase both generates hypermutation of the newly synthesized (-) strand DNA [29-31] and interferes with RT displacement along the template by a cytidine-deaminase independent mechanism [32,33].

Here, we show that the catalytic subunit of the cAMP-dependent protein kinase (PKA) packaged into HIV-1 viral particles acts as a cofactor for reverse transcription. Our previous observations have established that the Cα catalytic subunit of PKA packaged into viruses is required for viral infectivity [34]. The present study demonstrates that HIV-1 particles with reduced virus-associated PKA activity, display mature protein profile and have no apparent alteration in morphology. They contain wild type genomic RNA levels and reverse transcriptase activity, but are severely impaired for synthesis of (-)ssDNA and production of subsequent RT intermediates in infected cells. Using endogenous reverse transcription (ERT) experiments, we provide evidence that PKA is required for optimal RT reaction in cell free particles. These results provide new insight into the mechanisms of HIV-1 reverse transcription.

## Results

### Phenotypic characterization of HIV-1 particles produced from PKA-deficient cells

**Figure 1:**
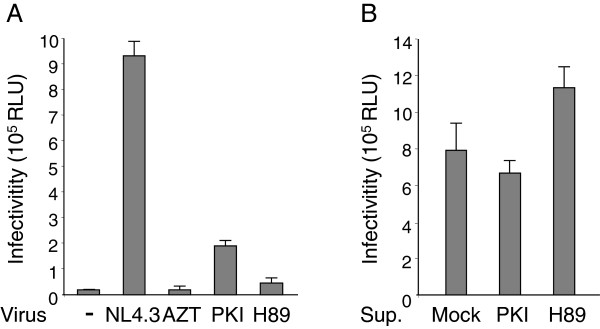
**Infectivity of H89-NL4.3 and PKI-NL4.3. (A)** Infectivity of normalized amounts of sucrose purified NL4.3, PKI-NL4.3 and H89-NL4.3 (10 ng p24 standardized to a final volume of 100 μL) was assayed in MAGIC-5B indicator cell lines. Control experiments consisted of mock infected cells (-) or NL4.3 infected cells maintained in the presence of 10 μM AZT. Infectivity was quantified by β-galactosidase activity in total cell lysate. Each value represents an average of three experiments performed in triplicate ± standard deviation. **(B)** Evaluation of viral samples contamination by co-purifying inhibitors. Supernatant (Sup.) of uninfected 293 cells maintained in medium alone (Mock) or cultured in the presence of H89 or Myr-PKI were subjected to gradient sucrose ultracentrifugation. Samples (100 μL) were then mixed with NL4.3 viruses (10 ng p24) produced from PKA proficient cells and the mixture was used to infect MAGIC-5B cells. Infectivity was measured as described in **(A)**.

The catalytic subunit of PKA is packaged into HIV-1 particles. We have previously demonstrated that cultivation of HIV-1 infected cells with H89, a chemical competitive inhibitor of ATP that blocks PKA activity, leads to the production of particles containing reduced virus-associated PKA activity and display poorly infectivity [34]. The present study was designed to extend these observations and to investigate the contribution of virus-associated PKA in HIV-1 life cycle. The number of encapsidated kinases identified so far in purified HIV-1 particles is limited, and PKA appears to be the unique packaged host-cell kinase susceptible to H89 inhibition (for review see [35]). Nevertheless, since kinase inhibitors generally display large inhibition spectra, we reconsidered our strategy and used genetic approaches, siRNA transfection and expression of dominant negative mutants, to extinguish PKA expression in HIV-1 producing cells. However, both approaches resulted only in partial inhibition of PKA in the HIV-1 producing cell (Additional file 1: Figure S1). According to this reproducible result, we rather conceived a strategy based on the use of Myr-PKI, a highly specific cell permeable peptide corresponding to the active domain of an endogenous PKA inhibitor [36]. HIV-1 particles were produced by transfection of the pNL4.3 molecular clone in 293T cells cultured in the presence of Myr-PKI using experimental procedures described elsewhere [34]. Similar experiments performed in the presence of H89 inhibitor were run in parallel. Concentrations of inhibitors used in these experiments were controlled to reduce significantly the cAMP-stimulated PKA activity in the producing cells using an *in vitro* kinase assay (Additional file 2: Figure S2A). Viral particles released from these cells were next subjected to sucrose cushion ultracentrifugation and normalized according to p24 content. In agreement with our previous observations [34], lysate of NL4.3 particles produced in absence of inhibitor efficiently phosphorylated a PKA peptide-substrate in *in vitro* kinase experiments (Additional file 2: Figure S2B). Addition of 20 μM cAMP to the reaction was unable to enhance substrate phosphorylation, confirming that HIV-1-associated PKA is incorporated into HIV-1 particles in an active form as previously reported [34]. In similar experimental conditions, normalized amounts of lysed viruses produced in the presence of Myr-PKI or H89 inhibitors, referred below as PKI-NL4.3 and H89-NL4.3 respectively, displayed reduced virus-associated PKA activity (Additional file 2: Figure S2B). Next, infectivity of virus preparations was tested using the MAGIC-5B indicator cell line expressing a β-galactosidase reporter gene under the control of HIV-1 LTR [37]. Infection levels were monitored by quantification of β-galactosidase activity in whole cell extracts. As presented in Figure 1A, infection of cells exposed to PKI-NL4.3 and H89-NL4.3 was approximately 8–10 times lower that observed from cells exposed to equivalent amounts of wild-type NL4.3 particles. The cAMP/PKA pathway is recognized to control HIV-1 transcriptional activity through TPA responsive elements (TRE)-like cAMP-responsive elements within the 5′-untranslated (U5) leader [38]. Inhibition of cellular PKA activity could therefore decrease LTR-driven transcriptional activity in MAGIC-5B cells. Accordingly, we evaluated the possible interference of any contaminating PKA inhibitor that would have co-sedimented with viral preparation. Mock-transfected cells were maintained in the presence of H89 or Myr-PKI inhibitors in conditions identical to those used for virus production. Then, supernatants were subjected to sucrose cushion ultracentrifugation, pellets were resuspended and mixed with NL4.3 viruses produced from PKA proficient cells. Ratio of inhibitor-containing supernatants to NL4.3 particles was chosen to mimic viral preparations used in Figure 1A. Then the mixtures were incubated with MAGIC-5B cells. As shown in Figure 1B, no significant difference in HIV-1-induced LTR-driven β-galactosidase reporter gene expression was observed for NL4.3 and viral preparations supplemented with Myr-PKI- or H89-containing supernatants. Accordingly, reduced infectivity observed for H89-NL4.3 and PKI-NL4.3 preparations in Figure 1A could reasonably not be ascribed to contamination of viral preparation with cosedimented inhibitor.

### H89-NL4.3 and PKI-NL4.3 display distinct phenotypes in primary human CD4^+^ lymphocytes and macrophages

**Figure 2:**
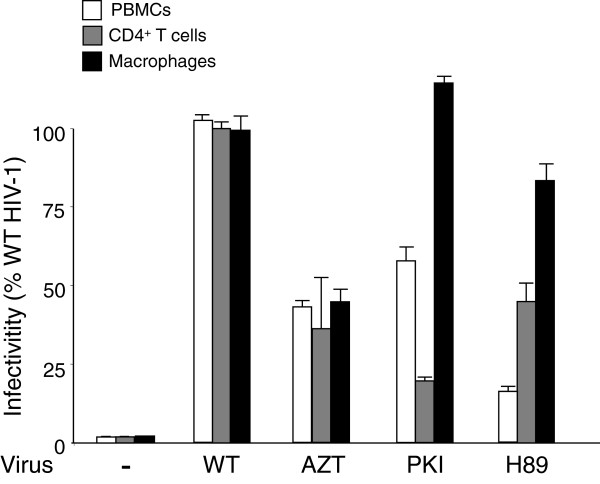
**Phenotypes of HIV-1 lacking PKA activity in primary human cells.** Infectivity of normalized amounts of sucrose purified NL4.3, PKI-NL4.3 and H89-NL4.3 (50 ng p24 standardized for 1x10^6^ cells) was assayed in PBMCs, CD4^+^ lymphocytes. Similar experiments were performed using primary monocyte-derived macrophages as target cells and NL81.A, PKI-NL81.A and H89-NL81.A viruses. At day 6 post-infection, HIV-1 DNA levels were monitored by qPCR amplification. Control experiments consisted of mock infected cells (-) or NL4.3- or NL81.A-infected cells maintained in the presence of 10 μM AZT. Data represent the average of duplicates ± SD and are expressed as percentages of wild-type conditions.

Next, we tested whether viral phenotypes observed using the MAGIC-5B indicator cell line were conserved in primary human cells. PBMCs were prepared from blood samples from uninfected donor and CD4^+^ lymphocytes or macrophages were isolated using magnetic beads-conjugated mAbs directed to cell surface antigens and magnetic field separation. After activation, cells were infected with normalized amounts of NL4.3, H89-NL4.3 or PKI-NL4.3. As attested by qPCR analysis, HIV-1 DNA levels were significantly reduced in PBMCs infected with H89-NL4.3 and PKI-NL4.3 as compared with copy number detected in cells infected with NL4.3 (Figure 2). A similar tendency was observed when CD4^+^ primary lymphocytes were processed in similar condition. Altogether, these data corroborate our previous observations [34] and indicate that NL4.3 viruses produced from cells with poor PKA activity are impaired for infectivity. To evaluate the role of HIV-1-associated PKA in primary macrophages, CCR5-tropic NL81.A HIV-1 viruses were produced by transfection of HEK 293T cells cultured in the presence PKA inhibitors using above described experimental procedures. When used to infect macrophages, H89-NL81.A and PKI-NL81.A behaved as wild type NL81.A, generating comparable retroviral cDNA levels. Altogether, these data indicate that HIV-1 produced from PKA-deficient cells display similar phenotypes in MAGIC-5B indicator cells and in primary CD4^+^ lymphocytes. Defects accounting for reduced viral replication in these cells were apparently efficiently compensated in macrophages since H89-NL81.A and PKI-NL81.A replicated at wild type levels in these cells.

### H89-NL4.3 and PKI-NL4.3 viruses are fully mature and normally assembled

**Figure 3:**
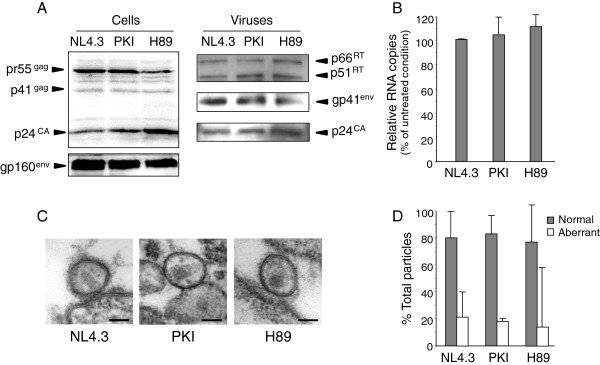
**Characterization of PKI-NL4.3 and H89-NL4.3 viral particles. (A)** 293T cells expressing HIV-1 NL4.3 were maintained in the presence of medium alone or supplemented with Myr-PKI (PKI) or H89 inhibitors. Normalized amounts of cells lysates (left panel) or sucrose cushion purified viruses (right panel) were sequentially probed with rabbit anti-RT or anti-gp41 sera or anti-p24 mAbs. **(B)** Genomic RNA in viral particles was quantified by qRT-PCR. Values are expressed as percentages of NL4.3 values ± SD. **(C)** NL4.3, PKI-NL4.3 and H89-NL4.3 viral particles were imaged by electron microscopy. Bar = 100 nm. **(D)** The number of fully mature and aberrant viruses was counted in each sample and expressed as a percentage of total observation (NL4.3 n = 57; PKI-NL4.3 n = 105; H89-NL4.3 n = 18). Error bars represent 95% confidence intervals.

In an attempt to investigate the nature of defects accounting for reduced infectivity of H89-NL4.3 and PKI-NL4.3 viruses, viral particles were next analyzed for protein content, maturation and assembly. Cell free particles or total extracts prepared from the corresponding producing cells were lysed and subjected to immunoblotting analysis. Samples were normalized according to total protein content for cell extracts and depending on the p24 concentration for viral particles preparations. Similar HIV-1 protein patterns were observed in cells cultured in absence or presence of PKA inhibitors. Regarding cell free particles, mature p24 was detected at similar levels in NL4.3, PKI-NL4.3 or H89-NL4.3 particles. Also, levels of mature RT subunits and gp41 incorporation were similar in all viral samples analyzed (Figure 3A). Real time reverse transcription PCR (qRT-PCR) analysis revealed that genomic RNA was packaged with similar efficiency in NL4.3, PKI-NL4.3 and H89-NL4.3 viruses (Figure 3B). Finally, electron microscopy imaging of individual particles revealed no visible difference in morphology, size or maturation between these viruses (Figure 3C and 3D). Accordingly, infectivity defects observed for NL4.3 viruses produced in the presence of Myr-PKI or H89 could be inferred neither to alteration of viral proteins incorporation or maturation, nor to impaired nucleic acid incorporation or to visible assembly modification.

### Virus-associated PKA is required for HIV-1 genome reverse transcription

**Figure 4:**
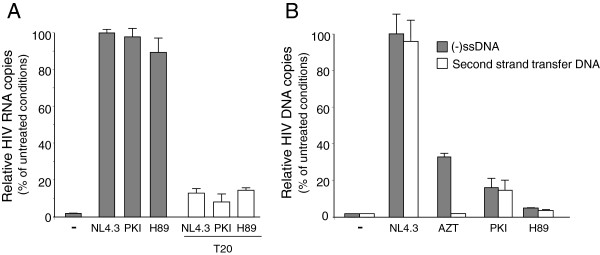
**Quantification of genomic RNA and reverse transcription intermediates in cells infected with NL4.3, PKI-NL4.3 or H89-NL4.3 viruses.** MAGIC-5B cells were infected with normalized amounts of NL4.3, PKI-NL4.3 or H89-NL4.3. Levels of genomic RNA **(A)** or (-)ssDNA or second strand transfer cDNA **(B)** in the infected cells were monitored by qRT-PCR or qPCR respectively. Control experiments consisted of mock infected cells (-) or NL4.3 infected cells maintained in the presence of 10 μM AZT. Values are expressed as percentage of NL4.3 conditions ± SD. Specificity of qRT-PCR detection of genomic RNA was controlled by preincubation of the cells for 30 min at 37°C in the presence of 1 μg/ml T20 fusion inhibitor before virus exposure.

Life cycle of H89-NL4.3 or PKI-NL4.3 viruses was next addressed by monitoring incoming genomic RNA in infected cells and quantifying cDNA intermediates synthesized during reverse transcription. First, levels of genomic RNA present in MAGIC-5B cells were quantified by qRT-PCR. Samples were prepared 1 hour after viral exposure to avoid sample contamination with *de novo* synthesized viral mRNAs and infections were performed in the presence of 10 μM AZT in order to prevent degradation of the RNA genome by the RT RNAse H activity during progression of the reverse transcription reaction. Similar RNA copy numbers were detected in cells infected with WT, H89-NL4.3 or PKI-NL4.3 viruses (Figure 4A). Preincubation of the cells with the T20 fusion inhibitor significantly reduced genomic RNA levels detected in the cells following infection with any virus tested, attesting for the specificity of our data. Because all viruses could enter target cells equally well, the rate and extent of reverse transcription performed by these viruses were analyzed by qPCR amplification of DNA intermediates 24 hours post-infection. In these experiments, viruses were treated with DNAse-I before addition to MAGIC-5B cells to remove any contaminant plasmid DNA in viral preparations. As shown in Figure 4B, the amount of (-)ssDNA detected in cells infected with H89-NL4.3 or PKI-NL4.3 was 5 to 15% that quantified in cells infected with NL4.3. A similar reduction was observed when the late RT product, second strand transfer DNA, was amplified. As expected, control consisting of cells infected with NL4.3 maintained in the presence of 10 μM AZT immediately after virus challenge showed reduced levels of both early and late reverse transcription intermediates. Altogether these data indicate that H89-NL4.3 and PKI-NL4.3 viruses efficiently infect their target cells, but are impaired for early reverse transcription.

### H89-NL4.3 and PKI-NL4.3 viruses display wild type intrinsic reverse transcriptase activity

**Figure 5:**
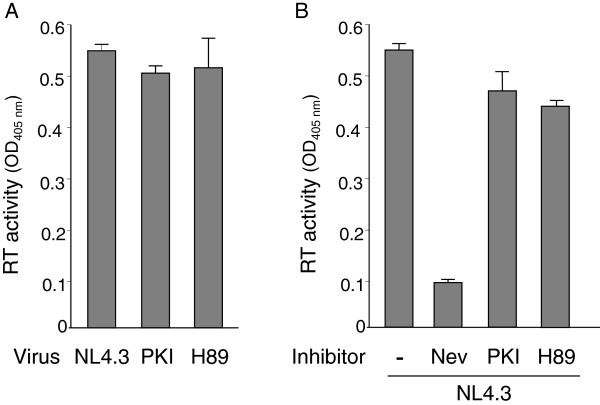
**Quantification of intrinsic RT activity contained in NL4.3, H89-NL4.3 and PKI-NL4.3 particles. (A)** Normalized amounts of sucrose-purified NL4.3, PKI-NL4.3 or H89-NL4.3 viruses were lysed and subjected to RT reaction in the presence of exogenous poly(A)/oligo dT template/primer hybrid and dNTPs. **(B)** RT reactions were performed with lysate of NL4.3 viruses incubated in the presence of H89 (100 μM) or Myr-PKI (50 μM) inhibitors. Addition of nevirapine (Nev) to the reaction mixture was used as a negative control. RT activity was quantified by ELISA as described in the Material and Methods section. Results are the mean of three separate experiments performed in triplicate ± standard deviation.

DNA synthesis defects evidenced for H89-NL4.3 and PKI-NL4.3 viruses reflect an impairment of the reverse transcription reaction. Therefore, we analyzed the enzymatic activity of reverse transcriptase packaged in H89-NL4.3 and PKI-NL4.3. Viral particles were lysed in the presence of NP-40 and incubated with a mixture of exogenous poly(rA)/oligodT template/primer hybrid and dNTPs. In these conditions, the RT activity, measured as the amount of dNTP incorporated into the template, was identical for normalized amounts of the three viruses (Figure 5A). To analyze further whether virus-associated PKA activity was required for activity of the reverse transcriptase, we repeated RT assays using lysate of NL4.3 viruses supplemented with inhibitory concentrations of Myr-PKI or H89. As shown in Figure 5B, RT-catalyzed cDNA synthesis from a synthetic template/primer was identical in presence or in absence of inhibitor. As expected, nevirapine, an inhibitor of RT, used in the same conditions almost completely inhibited the exogenous RT reaction. Accordingly, enzymatic activity of the HIV-1 reverse transcriptase does not require a functional PKA.

### Endogenous reverse transcription is impaired by PKA inhibitors

**Figure 6:**
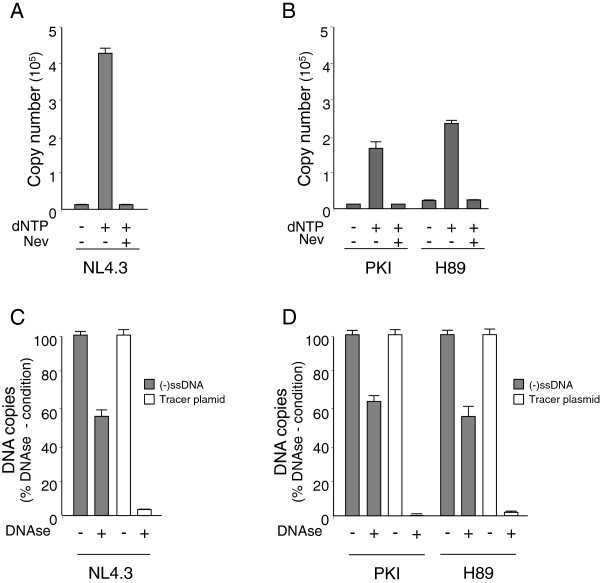
**Synthesis of (-)ssDNA in permeabilized NL4.3, H89-NL4.3 and PKI-NL4.3 viruses. (A)** Sucrose purified NL4.3 viruses permeabilized with 0.1 mM Triton X-100 were subjected to ERT reactions run for 16 h in the presence or absence of dNTPs. Control experiments consisted of incubation of viruses with dNTPs and 20 μg/mL nevirapine (Nev). (-)ssDNA copy numbers were determined in each sample by qPCR. Values are expressed as absolute copy numbers. Error bars indicate standard deviations (n = 4). **(B)** PKI-NL4.3 or H89-NL4.3 particles were subjected to ERT reactions as described in **(A)**. **(C)** and **(D)** Accessibility of ERT products synthesized in NL4.3, H89-NL4.3 and PKI-NL4.3 particles was monitored by addition 4 U DNase and 10^5^ copies of a tracer plasmid to the reaction mixture. After DNase inactivation and nucleic acids extraction, (-)ssDNA and tracer plasmid copy numbers were determined by qPCR. Values are expressed as a percentage of DNA levels detected in no DNAse reactions.

**Figure 7:**
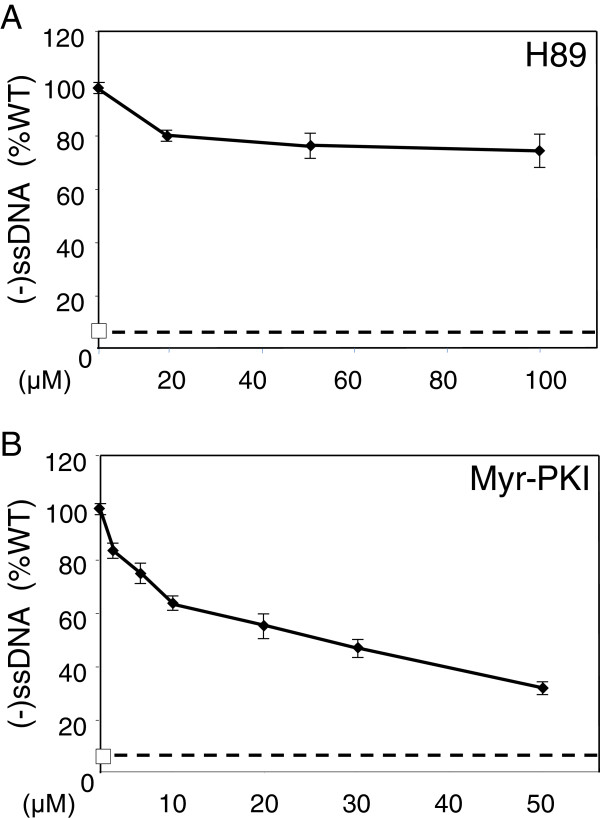
**Contribution of virus-associated PKA activity in endogenous reverse transcription.** NL4.3 viruses were subjected to ERT experiments run in the presence of dNTPs and increasing concentrations of H89 **(A)** or Myr-PKI **(B)**. For each reaction, nucleic acids were extracted and (-)ssDBNA production was quantified by qPCR amplification. DNA levels detected in ERT reactions performed in the presence of nevirapine are indicated by a dotted line. Data are mean values of duplicate experiments and are expressed as a percentage of WT conditions.

Reverse transcription can be initiated by addition of dNTPs into intact or mildly permeabilized HIV-1 particles, leading to the synthesis of FL DNA [12,16]. This experiment, referred to ERT (endogenous reverse transcription) assay merely serves as a model for understanding the reverse transcription occurring inside target cells. When performed using intact viral particles or virions incubated with low detergent concentrations, it gives access to the ability of the machinery contained in virions to produce proviral DNA using the RNA genome as a template and the tRNA^Lys3^ primer [14]. To decipher with the RT defect observed for PKI-NL4.3 and H89-NL4.3 viruses, viral particles were subjected to ERT reactions. Sucrose purified particles were permeabilized by addition of 0.1 mM Triton X-100 and incubated with dNTPs to allow synthesis of cDNA. Addition of low Triton X-100 concentrations (up to 0.1 mM) to the reaction mixture was reported to enhance levels of proviral DNA by permeabilizing the virion to nucleotides without destabilizing the viral particle [14]. Then, the reaction products were extracted and quantified by qPCR. Using these conditions, (-)ssDNA was detected at very low levels in NL4.3 particles maintained in absence of dNTPs and was drastically enhanced by addition of dNTPs (Figure 6A). Synthesis was abolished by addition of the membrane permeable inhibitor of reverse transcriptase nevirapine to the mixture, attesting for the specificity of the reaction. Next, PKI-NL4.3 and H89-NL4.3 viruses were subjected to ERT reactions using similar experimental conditions. When compared with NL4.3 viruses, (-)ssDNA synthesis was reduced by 44% and 27% for PKI-NL4.3 and H89-NL4.3 particles respectively (Figure 6B). Accordingly, viral particles produced in the presence of PKA inhibitors are less efficient at producing (-)ssDNA in ERT reaction. To examine whether such defects were linked to modification of the integrity of viruses engaged in the reaction, ERT experiments were repeated and followed by a DNAse digestion step of the final reaction mixture. A tracer plasmid (10^5^ copies) was added to the mix to control the efficiency of DNAse treatment. Then, total DNA present in the reaction was extracted and both proviral DNA synthesized in viral particles and tracer plasmid were monitored by qPCR. In our experimental conditions, levels of tracer plasmid detected by qPCR were reduced to background level by DNAse treatment, attesting for the efficiency of the digestion step (Figure 6C). In contrast, (-)ssDNA detected in NL4.3 particles following addition of dNTPs were only reduced by 40%, indicating that HIV-1 cDNA remained mostly inaccessible to degradation by DNAse. Accordingly, as previously demonstrated by others Triton X-100 used at a 0.1 mM concentration permeabilized the virions to nucleotides without totally disrupting the viral particles and allowed intraviral genome reverse transcription [14]. When applied to ERT products generated by Triton-permeabilized H89-NL4.3 or PKI-NL4.3 particles, DNAse I treatment reduced levels of (-)ssDNA and tracer plasmid in proportion similar to that observed for NL4.3 particles (Figure 6D). Therefore, the reduced capacity of H89-NL4.3 and PKI-NL4.3 viruses to produce (-)ssDNA in ERT experiments cannot be ascribed to drastic structural modification of the viral particles. Finally, to investigate whether the reduced efficiency of ERT observed in PKI-NL4.3 and H89-NL4.3 viruses was not due to any defect acquired during particles biogenesis, NL4.3 particles were subjected to ERT reaction performed in the presence of PKA inhibitors. The above-described experimental procedures were repeated starting from Triton X-100-permeabilized NL4.3 particles incubated in the presence of dNTPs, but reactions were supplemented with increasing concentrations of Myr-PKI or H89 before allowing viral DNA synthesis. As shown in Figure 7A and 7B, (-)ssDNA synthesis decreased as the concentration of H89 or Myr-PKI increased in the reaction (Figure 7A and 7B). The addition of 100 μM H89 lowered ERT activity by 25% for H89 and Myr-PKI decreased (-)ssDNA synthesis by 70% when added at 50 μM. Accordingly, H89 and Myr-PKI likely inhibit an intraviral process in NL4.3 particles required for optimal efficiency of the early reverse transcription reaction.

## Discussion and conclusion

The aim of this study was to decrypt the role played by HIV-1-associated PKA in virus infectivity. To this end we took advantage of our capacity to produce HIV-1 particles containing reduced PKA activity. This goal was previously achieved by mean of the cell-permeable H89 inhibitor of PKA [34]. To improve the specificity of our experimental model, we initially focused on the use of siRNA targeting mRNA encoding the Cα catalytic subunit of PKA. Unfortunately, despite reducing significantly intracellular PKA levels, a basal expression remained in the producing cells that accounted for residual PKA incorporation into virions. These viral particles were approximately 40% less infectious than normalized amounts of wild type HIV-1. Similarly, ectopic expression of a D324G mutant of PKA-RIα regulatory subunit, that is unable to dissociate from PKA catalytic subunit, resulted in an intermediate phenotype (Additional file 1: Figure S1). Accordingly, this strategy was abandoned in favour of the use of chemical inhibitors whose capacity to inhibit PKA rely on distinct mechanisms. Myr-PKI is a myristoylated peptide which contains a PKA substrate consensus sequence with the serine phosphor-acceptor site replaced by alanine [36]. This peptide inhibits PKA enzymatic activity and displays high selectivity by docking to the substrate binding site of the kinase [39]. The parallel use of Myr-PKI with that of H89, a competitive inhibitor of ATP during kinase activation, therefore limits the risk to focus on off target effects resulting from wide spectrum kinase inhibitory properties.

Here we found that HIV-1 particles produced from virus-expressing cells cultured in the presence of H89 or Myr-PKI concentration effective at inhibiting cellular PKA activity were fully mature and displayed no apparent assembly defect. Particularly, they contained expected levels of mature p51 and p66 RT subunits and intrinsic RT activity was unaffected as evidenced in exogenous RT assays performed using a synthetic poly(rA)/oligodT template. Moreover, genomic RNA content was comparable to that of wild type NL4.3 particles. H89-NL4.3 ad PKI-NL4.3 viruses efficiently infected targets cells as supported by the levels of incoming genomic RNA in cell extracts. However, these viruses displayed reduced infectivity and were unable to reverse transcribe the viral genome into FL proviral DNA. Indeed, viruses were defective for (-)ssDNA production indicating that an early step of the reverse transcription process was impaired. Testing of intrinsic RT activity associated with H89-NL4.3 and PKI-NL4.3 viruses together with experiments based on incubation of wild type RT in the presence of chemical inhibitors revealed that reverse transcriptase enzymatic activity was unaffected by PKA perturbators. Nevertheless, H89-NL4.3 and PKI-NL4.3 viruses displayed a reduced capacity to produce (-)ssDNA in ERT, when incubated with dNTPs in conditions maintaining the integrity of viral particles. Accordingly, such viruses were defective for an intraviral process required for optimal reverse transcription. Interestingly, ERT could be inhibited in NL4.3 particles by addition of PKA inhibitors to the reaction mixture. Therefore, the possibility that the decreased DNA synthesis evidenced for H89-NL4.3 and PKI-NL4.3 viruses in ERT experiments is consecutive to an interference with intracellular assembly in cell cultured in the presence of PKA inhibitors can be reasonably rejected. As PKA is incorporated in an active form in HIV-1 particles [34], our data support that the virus-associated kinase is required either to produce a functional reverse transcription complex in the viral particle or to create the appropriate microenvironment required for optimal reverse transcription.

The exact contribution of PKA in proviral DNA synthesis needs further investigation. A number of studies have indicated that p51 and p66 subunits of HIV-1 reverse transcriptase are post-translationally modified in virions, infected cells and RTCs [40,41]. Such modifications enhanced reverse transcription efficiency and progression. However, the contribution of PKA in RT phosphorylation was rejected [42]. Nevertheless, it remains possible that virus-associated PKA could modify a virus-incorporated HIV-1 encoded protein assisting reverse transcription. In this context, the role of Nef and Vpr, two PKA-phosphorylated proteins both known to be required for proviral DNA synthesis, needs to be investigated [43,44]. A possible regulation of RT through modulation of the APOBEC3G/Vif interplay should also be considered as PKA-mediated phosphorylation of APOBEC3G can regulate the interaction between A3G and Vif [45]. In addition, the capacity of PKA to phosphorylate the HIV-1 p24 protein *in vitro* and to interact with PKA *in vivo* has to be kept in mind [34]. Indeed, substitution mimicking phosphorylation of serine residues identified as phospho-acceptor sites either in the p24 interdomain linker or in helix 9, a dimerization interface required for core assembly, inhibits reverse transcription [46]. Such mutations rendered the HIV-1 core unstable and generated mild assembly defects. According to these data, and to data presented herein, HIV-1-associated PKA could fulfill a complex role during post-entry steps of HIV-1 infection through the phosphorylation of multiple targets thereby creating a microenvironment that favours the initiation and the progression of the reverse transcription. A series of experiments is under progress to test these hypotheses.

Finally, phenotypes observed for H89-NL4.3 and PKI-NL4.3 viruses differed according to the target cell. RT defects observed in indicator cells and CD4^+^ lymphocytes were efficiently compensated in macrophages, allowing synthesis of wild type levels of proviral DNA. Suppressing host cell cAMP-dependent PKA activation by β-chemokines (MIP-1α, MIP1-β and RANTES) was previously reported to inhibit HIV-1 cDNA synthesis in primary CD4^+^ lymphocytes but not in macrophages [47]. In agreement with these observations, our data indicate that HIV-1-associated PKA might exert differential effects in primary lymphocytes and macrophages and that post-entry steps may significantly differ in these two cell types.

Overall, a complex interplay between PKA and HIV-1 infection has been reported in the literature. Increased intracellular levels of cAMP and constitutive activation of PKA were measured in cells from HIV-1 infected patients [48]. Such defect correlated with T cells dysfunction and immunodeficiency [48-50]. To the opposite, reduction of intracellular cAMP levels or PKA agonists restores immune responses in T cells isolated from HIV-1 infected patients through the inhibition of the cAMP/PKA pathway [48,51]. At the cell level, modulation of the cAMP/PKA pathway was reported to enhance HIV-1 replication at multiple steps, including LTR-driven HIV genes transcription [47,52,53] and proviral DNA synthesis [47,52,53]. Here, we showed that incorporation of an active PKA determines the efficiency of early steps of reverse transcription in a new target cell. In light of these observations, both the PKA-dependent pathway in the target cell and virus-associated PKA represent key players in HIV-1 replication and pathogenesis. This model agrees with the results of recent meta-analysis of genome-wide studies that identified PKA as an important host factor in early HIV-1 replication [26].

## Methods

### Cells

Human embryonic kidney 293T cells and the MAGI-5B indicator cell line [37] were cultured in Dulbecco’s modified Eagle’s medium (Life Technologies, Inc.) supplemented with 10% fetal calf serum (Cambrex), 100 units/mL penicillin, 100 μg/mL streptomycin. Peripheral blood mononuclear cells (PBMCs) from healthy donors were isolated from buffy coats by Ficoll-Hypaque gradient centrifugation. CD14^+^ monocytes were isolated with anti-human CD14-coated magnetic beads (Miltenyi Biotec) according to manufacturer recommendations and differentiated into macrophages by incubation for 5 days in complete medium (RPMI 1640-10% fetal calf serum) supplemented with hM-CSF (Immunotools). CD4^+^ T cells were isolated from CD14 - peripheral blood lymphocytes with a CD4^+^ T-cell enrichment kit (Stem Cell Technologies). Cells were then resuspended in RPMI 1640 medium supplemented with 10% heat-inactivated fetal calf serum and stimulated for 3 days with 5 μg/ml phytohemagglutinin in the presence of 20 U/ml of interleukin-2 (Roche).

### Viral stock production

Viral stocks were generated as previously described [34]. 293T cells (5x10^6^) were transfected with 30 μg of pNL81.A (kindly provided by W.C. Greene) or pNL4.3 DNA (AIDS Research and Reference Reagent Program, Division of AIDS, NIAID, NIH) using calcium phosphate precipitation. Twenty-four hours post transfection, the cells were extensively washed and cultured for an additional 16 h in medium alone or supplemented with 100 μM H89 (Sigma-Aldrich) or with 50 μM Myr-PKI peptide (Myr-_14_GRTGRRNAI_22_-NH2) (Sigma-Aldrich). Virus-containing supernatants were collected, filtered onto 0.45 μm membranes. All viral preparations were purified by centrifugation through 20% sucrose cushion at 25,000 rpm for 2.5 h at 4°C in a Sw32Ti rotor (Beckman Coulter, France). The pellets were resuspended in PBS, aliquoted and stored at -80°C. Viral stocks were normalized according to p24 content using an anti-p24 Enzyme-linked Immunosorbent Assay (Ingen, France).

### Viral infectivity assays

MAGI-5B cells which stably express the β-galactosidase reporter gene cloned downstream of the HIV-1 LTR promoter were platted at 8 × 10^3^ cells/100 μL in 96 wells plates. The cells were exposed to viral solutions normalized according to p24 content. 48 hours post-infection, virus infectivity was monitored by measurement of β-galactosidase activity from the cell lysates as previously described using the Galactostar β-galactosidase assay kit (Applied Biosystem). Luminescence was recorded using a Centro XS3 LB960 luminometer (Berthold, France). Values were normalized according to protein content of the cell extract (BCA assay kit, Pierce). T20 fusion inhibitor was obtained from the AIDS Research and Reference Reagent Program (Division of AIDS, NIAID, NIH).

### Immunoblotting analysis

Cell or sucrose-purified virions were solubilized in RIPA buffer and separated on a 12.5% SDS-PAGE. Proteins were transferred to PVDF membrane (Millipore) and revealed using a rabbit polyclonal serum directed to RT (kindly provided by J. L. Darlix, ENS, Lyon, France) or mAbs raised against CA (Biogenesis) or anti-gp41 (Fitzgerald Inc.). Secondary antibodies conjugated to horseradish peroxidase were revealed by enhanced chemiluminescent detection (Pierce Biotechnology Inc.).

### Electron microscopy analysis

Virus-producing cells were processed for thin-layer electron microscopy as described elsewhere [34]. Briefly, the cells were fixed *in situ* with 2.5% glutaraldehyde in cacodylate buffer (pH 7.4) for 60 min at 4°C. Cells were then post-fixed with 2% osmium tetroxide, washed in cacodylate buffer containing 0.5% tanic acid, and embedded in epon (Embed-812, Electron Microscopy Sciences Inc.). Preparations were examined with a Hitachi H.7100 transmission electron microscope (CRIC, University Montpellier 1, France).

### qPCR analysis of genomic RNA and proviral DNA

Total RNA isolated from cells challenged with HIV for 1 h or genomic RNA contained in HIV-1 particles (100 ng p24) was extracted with Tri-Reagent (Sigma, France) and subjected to qRT-PCR analysis as previously described [54]. Briefly, a poly(dT) olygonucleotide was used as RT-primer and subsequent qPCR was performed with a the SYBR® Green PCR Master Mix (Roche, France) on the RotorGene system (Labgene, France). A 90 bp fragment in the R-U5 region from the HIV-1 RNA was amplified using 5′-AGCAGCTGCTTTTTGCCTGTA-3′ and 5′-AAGCAGTGGGTTCCCTAGTTAG-3′ oligonucleotides. A standard curve was generated from 10^2^ to 10^6^ copies of pNL4.3 plasmid. Each assay was accompanied by controls without reverse transcriptase (DNA contamination levels <1% of HIV-1 RNA). The copy numbers of HIV-1 genomic RNA in cell samples were normalized to that of the GAPDH mRNA quantified in parallel as endogenous control.

For proviral DNA analysis, total DNA was extracted from cells infected for 24 h with DNAse I-treated virus, using the DNeasy extraction kit (Qiagen). In order to check for the absence of contaminating plasmid (pNL4.3 or pNL8.1A) in samples, DNA preparation were tested with a primer pair that specifically amplifies the pUC region of the molecular clone as previously reported [46], and all samples found positive were discarded. HIV-1 DNA synthesis was then monitored by qPCR as described elsewhere [46]. Primers used for amplification were: (-)ssDNA: #73: 5′-ACACAACAGACGGGCACACAC-3′ and #187: 5′- GGTCTCTCTGGTTAGACCA-3′; second strand transfer DNA #169: 5′-AGCAGCTGCTTTTTGCCTGTA-3′ and MA: 5′-CCTGCGTCGAGAGATCTCCTCGG-3′.

### Analysis of intrinsic reverse transcriptase activity

Exogenous reverse transcription assays were performed to measure the enzymatic activity of the RT contained in lysate of viral particles (3 ng p24) when incubated in the presence of synthetic poly(rA)-oligo(dT) template and primer using the Reverse transcriptase assay colorimetric kit (Roche, France).

### Endogenous reverse transcription assays (ERT)

Proviral DNA synthesis in assembled viral particles permeabilized with reduced amounts of detergent was determined in ERT assays. This assay is based on the use of endogenous RNA genome as a template and that of the endogenous tRNA^Lys3^ as a primer. Sucrose cushion-purified virus particles (normalized according to p24 content to 10 ng) were incubated for 18 h at 37°C in the presence of 0.1 mM Triton X-100, 10 mM Tris, pH 7.4, 10 mM MgCl_2_, and 200 μM of each dNTP in a final volume of 50 μL. Nucleic acids were then extracted with DNeasy extraction columns (Qiagen) and eluted in 200 μL of water. RT products were quantified by qPCR amplification using 2.5 μL as template. As a control, the same mix was prepared for each sample but without dNTPs and a no-nucleotide control reaction mixture was always included. Accessibility of reverse transcription products to degradation after the permeabilization of virions with detergent was assayed by addition of 4U DNase I (Life Technologies) to the ERT products and incubation for 30 min at 37°C before nucleic acids extraction.

### Ethical approval

Primary human cells used for this work were obtained from the French blood establishment (EFS, Toulouse, France) in accordance with international ethical principles and French national law under declaration N° DC-2008-207 to the French Ministry of Research and Higher Studies.

## Abbreviations

PKA: cAMP-dependent protein kinase; HIV-1: Human immunodeficiency virus type 1; PBS: Primer binding site; RT: Reverse transcriptase; RTC: Reverse transcription complex; ERT: Endogenous reverse transcription; (-)ssDNA: minus strand strong stop DNA; dNTPs: deoxynucleotides; TRE: TPA-responsive element; LTR: Long terminal repeat; CypA: Cyclophilin A; AZT: Azidothymidine; qPCR: quantitative PCR; VSV-G: Vesicular stomatitis virus glycoprotein G.

## Competing interests

The authors declare that they have no competing interest.

## Authors’ contributions

CG, PE and SB performed the experiments. BG performed the electron microscopy studies. NC and LB designed the experiments, analyzed the data and wrote the paper. All authors read and approved the final manuscript.

## Supplementary Material

Additional file 1: Figure S1 siRNA against PKA-Cα or expression of dominant negative RI-α regulatory subunit in the producer cells reduce infectivity of NL4.3 particles. (A) MAGIC-5B cells were transfected with 50 nM control siRNA (siC) or siRNA against PKA Cα subunit (siPKA) (Cell Signaling Technology) using Interferin transfection reagent (Polyplus Transfection). Seventy-two hours after transfection, the culture was infected with HIV-1 NL4.3 and viral particles released in supernatants during the next 12 h were collected. Infectivity of virus preparations (20 or 50 ng p24 standardized to a final volume of 100 μl) was assayed in MAGIC-5B indicator cell lines. Mock infected cells (-) are shown as control. Each value represents the mean of duplicate experiments ± standard deviation. (B) Expression of PKA-Cα in virus-producing cells was monitored over time by immunoblot analysis. Proteins levels loaded in each line were monitored by incubation with anti-tubulin mAbs (Santa-Cruz Biotechnology, Inc.). (C) 293 T cells expressing the pNL4.3 HIV-1 molecular clone were transfected with the promoter inducible pPKA RIα^D324G^ plasmid encoding a dominant negative (DN) regulatory subunit unable to release PKACα [55] (kindly provided by Pr. B. Schimmer, University of Toronto) or with an empty vector (Mock) and culture in the presence of 100 μM Zn^2+^ for 24 h to induce transgene expression. Virions released in culture supernatant were normalized to 10 ng p24 and used to infect MAGIC-5B cells. Infectivity was determined by quantification of reporter gene expression in cell lysates. PKA activity in the producing cells was determined using the MESACUP protein kinase assay kit (MBL, Ltd., Nagoya, Japan). Values are expressed as percentage of mock conditions. Click here for file

Additional file 2: Figure S2 PKA is incorporated in an active form in NL4.3 but not in PKI-NL4.3 or H89-NL4.3 particles. (A) PKA activity in lysate of HIV-1-producing 293 T cells cultured in medium alone or in the presence of H89 or Myr-PKI was determined using the MESACUP protein kinase assay kit (MBL, Ltd., Nagoya, Japan). Addition of cAMP (20 μM) to the reaction mixture was used to stimulate kinase activity. (B) Normalized amounts of purified NL4.3, PKI-NL4.3 and H89-NL4.3 viruses were lysed and analyzed for PKA activity. NL4.3-associated kinase activity remained unchanged when 20 μM cAMP was added to the reaction mixture but was reduced by addition of H89 (20 μM) or Myr-PKI (10 μM) to the reaction mix, attesting that PKA is incorporated into HIV-1 particles in an active form. In these experimental conditions, kinase activity associated with normalized amounts of PKI-NL4.3 or H89-NL4.3 lysates was significantly reduced as compared with that detected from NL4.3 particles. Click here for file
