# Supplementary figures and images for: HIV-1-associated PKA acts as a cofactor for genome reverse transcription
Source: Retrovirology. 2013 Dec 17;10:157. doi: 10.1186/1742-4690-10-157 (PMC3880072; doi:10.1186/1742-4690-10-157)

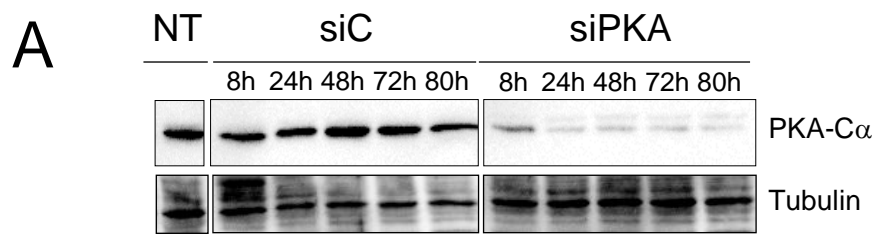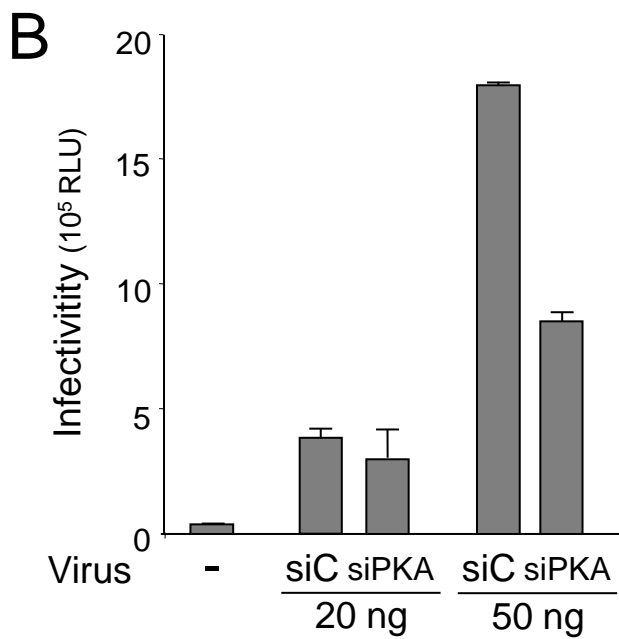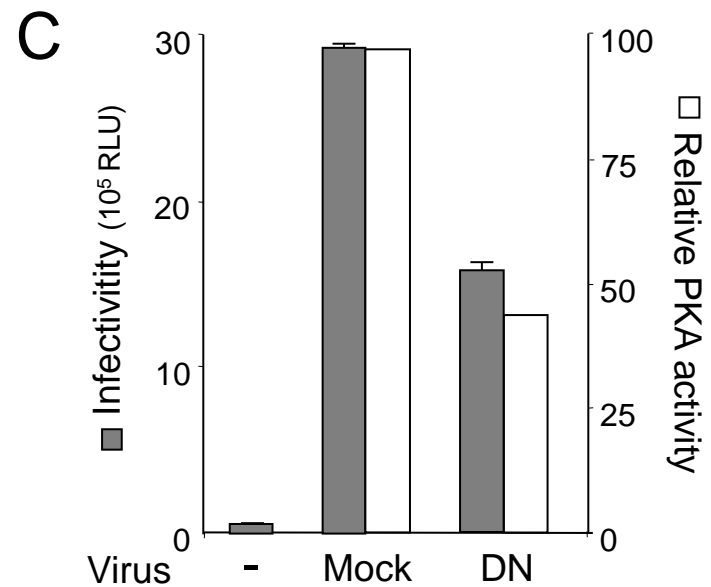

Supplement: Additional file 1: Figure S1 — siRNA against PKA-Cα or expression of dominant negative RI-α regulatory subunit in the producer cells reduce infectivity of NL4.3 particles. (A) MAGIC-5B cells were transfected with 50 nM control siRNA (siC) or siRNA against PKA Cα subunit (siPKA) (Cell Signaling Technology) using Interferin transfection reagent (Polyplus Transfection). Seventy-two hours after transfection, the culture was infected with HIV-1 NL4.3 and viral particles released in supernatants during the next 12 h were collected. Infectivity of virus preparations (20 or 50 ng p24 standardized to a final volume of 100 μl) was assayed in MAGIC-5B indicator cell lines. Mock infected cells (-) are shown as control. Each value represents the mean of duplicate experiments ± standard deviation. (B) Expression of PKA-Cα in virus-producing cells was monitored over time by immunoblot analysis. Proteins levels loaded in each line were monitored by incubation with anti-tubulin mAbs (Santa-Cruz Biotechnology, Inc.). (C) 293 T cells expressing the pNL4.3 HIV-1 molecular clone were transfected with the promoter inducible pPKA RIαD324G plasmid encoding a dominant negative (DN) regulatory subunit unable to release PKACα [55] (kindly provided by Pr. B. Schimmer, University of Toronto) or with an empty vector (Mock) and culture in the presence of 100 μM Zn2+ for 24 h to induce transgene expression. Virions released in culture supernatant were normalized to 10 ng p24 and used to infect MAGIC-5B cells. Infectivity was determined by quantification of reporter gene expression in cell lysates. PKA activity in the producing cells was determined using the MESACUP protein kinase assay kit (MBL, Ltd., Nagoya, Japan). Values are expressed as percentage of mock conditions. [file 1742-4690-10-157-S1.pdf]

**A**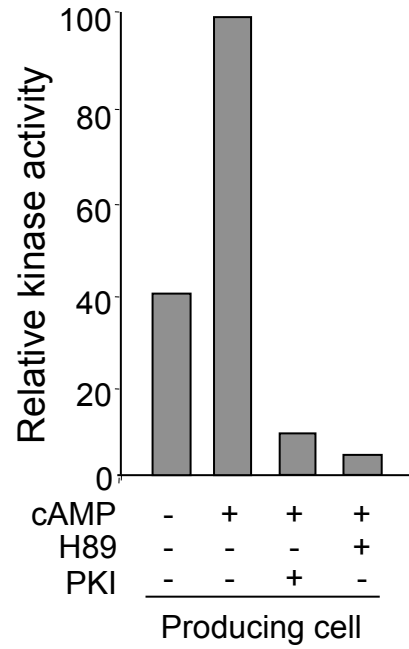**B**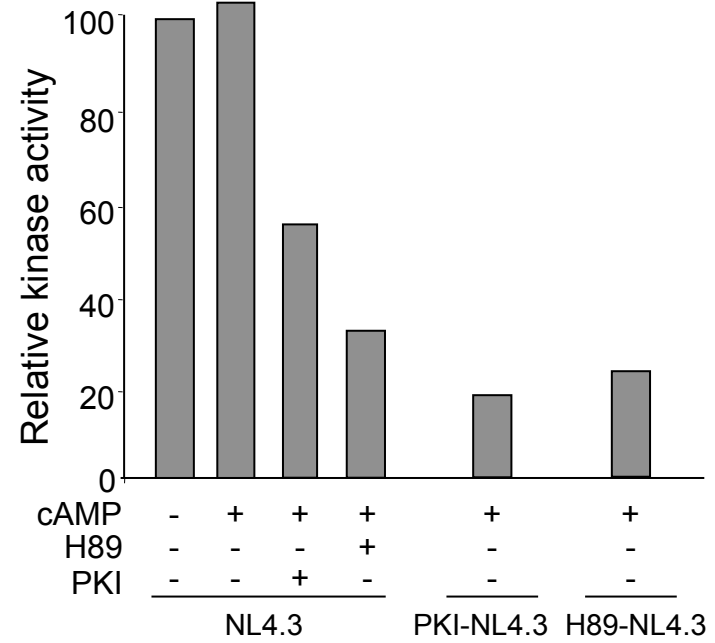

Supplement: Additional file 2: Figure S2 — PKA is incorporated in an active form in NL4.3 but not in PKI-NL4.3 or H89-NL4.3 particles. (A) PKA activity in lysate of HIV-1-producing 293 T cells cultured in medium alone or in the presence of H89 or Myr-PKI was determined using the MESACUP protein kinase assay kit (MBL, Ltd., Nagoya, Japan). Addition of cAMP (20 μM) to the reaction mixture was used to stimulate kinase activity. (B) Normalized amounts of purified NL4.3, PKI-NL4.3 and H89-NL4.3 viruses were lysed and analyzed for PKA activity. NL4.3-associated kinase activity remained unchanged when 20 μM cAMP was added to the reaction mixture but was reduced by addition of H89 (20 μM) or Myr-PKI (10 μM) to the reaction mix, attesting that PKA is incorporated into HIV-1 particles in an active form. In these experimental conditions, kinase activity associated with normalized amounts of PKI-NL4.3 or H89-NL4.3 lysates was significantly reduced as compared with that detected from NL4.3 particles. [file 1742-4690-10-157-S2.pdf]
